# Association between physical activity, nutritional status and cognitive performance among school children in southern Tanzania

**DOI:** 10.3389/fpubh.2025.1552215

**Published:** 2025-06-10

**Authors:** Fahad S. Mwakalebela, Elihaika G. Minja, Yohana A. Mwalugelo, Erick Killel, Rehema K. Rajab, Getrud J. Mollel, Winifrida Mponzi, Honorati Masanja, Fredros O. Okumu, Christin Lang, Markus Gerber, Jürg Utzinger, Kurt Z. Long, Efraim M. Kosia, Ester Elisaria, Marceline F. Finda

**Affiliations:** ^1^Department of Environment Health and Ecological Science, Ifakara Health Institute, Ifakara, Morogoro, Tanzania; ^2^Department of Global Health and Biomedical Sciences, School of Life Sciences and Bio-Engineering, The Nelson Mandela African Institution of Science and Technology (NM-AIST), Arusha, Tanzania; ^3^Swiss Tropical and Public Health Institute, Allschwil, Switzerland; ^4^University of Basel, Basel, Switzerland; ^5^Department of Community Health and Nutrition, Tanzania Food and Nutrition Center, Dar es Salaam, Tanzania; ^6^Department of Pediatric and Child Nutrition, Muhimbili University of Health and Allied Sciences, Dar es Salaam, Tanzania; ^7^School of Biodiversity, One Health and Veterinary Medicine, University of Glasgow, Glasgow, United Kingdom; ^8^Department of Sport, Exercise and Health, University of Basel, Basel, Switzerland

**Keywords:** cognitive performance, health, nutritional status, physical activity, school children, Tanzania, wellbeing

## Abstract

**Background:**

Physical activity is pivotal in promoting overall health and wellbeing, improving brain function and cognitive performance, and reducing risk of excessive weight and non-communicable diseases. Despite these benefits, physical inactivity among children and adolescents remains a global concern, particularly in low- and middle-income countries (LMICs). Only few studies have explored the association between physical activity, nutritional status, and cognitive performance in LMICs like Tanzania. This study assessed these associations among school children in a rural setting in southern Tanzania.

**Methods:**

Physical activity was assessed using an actigraphy device that assessed 7-day average of physical activity among school children aged 6–13 years. Cognitive performance was assessed via the Flanker task, which assessed the executive domain of cognitive functions, specifically information processing and inhibitory control. Weight was determined using Tanita digital scale, and height was measured using measuring board. A multinomial logistic regression model and a gamma generalized linear model with a log link function were used to examine the association between physical activity, nutritional status, and cognitive performance.

**Results:**

Among 678 children who participated in the baseline assessment, 77.9% had normal weight, 14.3% were underweight, 5.5% were overweight, and 2.4% were obese. Most (92.6%) of the children engaged in at least 60 min per day of moderate-to-vigorous physical activity (MVPA) as per WHO recommendations. Children who did not meet the recommended MVPA level were nearly three times more likely to be obese compared to those who did. No statistically significant associations were found between physical activity and cognitive performance, or between nutritional status and cognitive performance.

**Conclusion:**

This study highlights a high prevalence of normal weight and adherence to WHO-recommended MVPA levels among school children in southern Tanzania. However, children not meeting MVPA criteria were more likely to be obese, indicating the importance of physical activity in maintaining a healthy weight. The lack of association between physical activity or nutritional status and cognitive performance, indicates that there may be additional factors that influence cognitive outcomes. Further research is needed to explore these interactions, particularly in rural LMIC settings.

## Introduction

Physical activity plays a crucial role in promoting overall health and wellbeing across all age groups (1, 2). It offers several health benefits, including improved brain health (3, 4), effective weight management (5, 6), reduced risk of non-communicable diseases (NCDs) (7), stronger bones and muscles (8, 9), and improved everyday functionality (10, 11). The World Health Organization (WHO) highlights that physical activity among school children has positive impact on their mental health, school attendance, classroom behavior, memory, concentration, and academic achievement (12). On the other hand, insufficient physical activity levels are a risk factor for NCDs (13–15), leading to poor health outcomes in later life stages (16).

The WHO classifies physical activity into three intensity levels: light, moderate, and vigorous physical activity. This classification is essential for assessing adherence to recommended physical activity guidelines, which, for children, specify engaging in moderate-to-vigorous physical activity (MVPA) for at least 60 min per day (17). Alarmingly, more than 80% of children and adolescents globally fail to meet the recommended physical activity levels (12). In low- and middle-income countries [LMICs], 85% of children and adolescents aged 11–17 years are insufficiently physically active (18). In Tanzania, 78% of boys and 86% of girls in this age range do not meet recommended levels of MVPA (19).

Research demonstrates a strong correlation between sufficient physical activity and improved nutritional status. Regular physical activity increases calorie expenditure, regulates appetite, and enhances nutrient absorption, all of which contribute to maintaining a healthy body weight (20, 21).

Nutritional status refers to the condition of an individual’s health as influenced by the intake and utilization of nutrients (22). It is typically categorized into undernutrition, normal nutrition, and over nutrition (23). Globally, approximately 42 million children are overweight, with over 35 million living in LMICs (24). The prevalence of these nutritional statuses varies significantly between high-income countries and LMICs (25). However, studies show that overnutrition is on the rise in LMICs due to dietary and lifestyle changes, with reported overweight and obesity rates exceeding 10% across Africa (26). On the other hand, undernutrition, including stunting, wasting, and micronutrient deficiencies, remains prevalent in LMICs due to factors such as poverty, food insecurity, and limited access to healthcare (27). In Tanzania, the 2021 School Malaria and Nutrition Survey (SMNS) reported that 25.0% of school children aged 5–16 years were stunted, 11.7% were underweight, 11.0% were thin, whereas 5% were overweight or obese (28).

The impact of nutritional status extends beyond physical health, influencing cognitive development and performance, particularly in children. Emerging evidence highlights a significant association between nutritional status and cognitive performance, particularly in executive functions such as inhibitory control, working memory, and cognitive flexibility (29, 30). Malnutrition, especially during critical periods of brain development, can negatively impact cognitive abilities, leading to deficits in learning, memory, and executive functions (29, 31). Moreover, overnutrition, particularly obesity, has been linked to impaired cognitive performance, possibly due to inflammatory processes and metabolic dysregulation (32, 33). These associations underscore the importance of maintaining optimal nutritional status to support children’s cognitive development and academic achievement (34, 35).

Similarly, regular physical activity has been linked to improved cognitive performance among school children (20, 36, 37). Studies have shown that sufficiently active children present with enhanced cardiovascular efficacy, leading to increased oxygen delivery to the brain subsequently leading to improved cognitive performance (20, 36, 37). Overall, cognitive performance refers to mental processes such as thinking, learning, memory, and problem-solving (38, 39). Executive functions represent a specific type of cognitive performance, which describes higher-order cognitive abilities. Executive functions are multi-dimensional, including inhibitory control, working memory, and cognitive flexibility (40). Inhibitory control, considered by some authors as the critical component of executive functions (41, 42), involves the ability to regulate thoughts and actions, particularly in the face of distractions or temptations. This dimension is in the focus of our study due to its critical role in cognitive development and behavior regulation.

Recent studies have demonstrated that inhibitory control significantly predicts future academic success and wellbeing. Children with better self-control experience improved health, financial stability, and contribute to public safety in adulthood (43). Moreover, research suggests that inhibitory control, and self-control in general, may outweigh intelligence in determining life outcomes (44). Enhancing children’s inhibitory control capacity is crucial, as it can lead to better academic performance, healthier lifestyle choices, and improved social interactions. There is a paucity of studies on higher-order cognitive abilities in LMICs, which limits understanding of their estimates.

However, higher-order cognitive abilities seem to vary globally, as they are influenced by factors such as education, health, and socioeconomic status (45). Understanding these dynamics and their implications is crucial for identifying areas where interventions can improve cognitive outcomes and overall development (46, 47). While extensive evidence exists on the effects of physical activity on the health and wellbeing of children and adolescents in high-income countries and urban settings (2, 48–56), the same is not the case for rural settings in LMICs (36, 57). To address this gap, this study aimed at determining the association between physical activity, nutritional status, and higher-order cognitive abilities of school children in four public primary schools in rural southern Tanzania. The study utilized data collected through a larger cluster randomized controlled trial (RCT) known as the KaziAfya study, which investigated the effects of school-based physical activity and multi-micronutrient supplementation (MMS) on overall health and wellbeing of school children living in the Town Council in Southern Tanzania, conducted between 2019 and 2021 (58). The main research questions are as follows: (1) what is the link between nutritional status and physical activity? (2) What is the link between physical activity and higher-order cognitive abilities? (3) What is the link between nutritional status and higher-order cognitive abilities?

## Methods

### Study area, study design, and procedures

**Figure 1:**
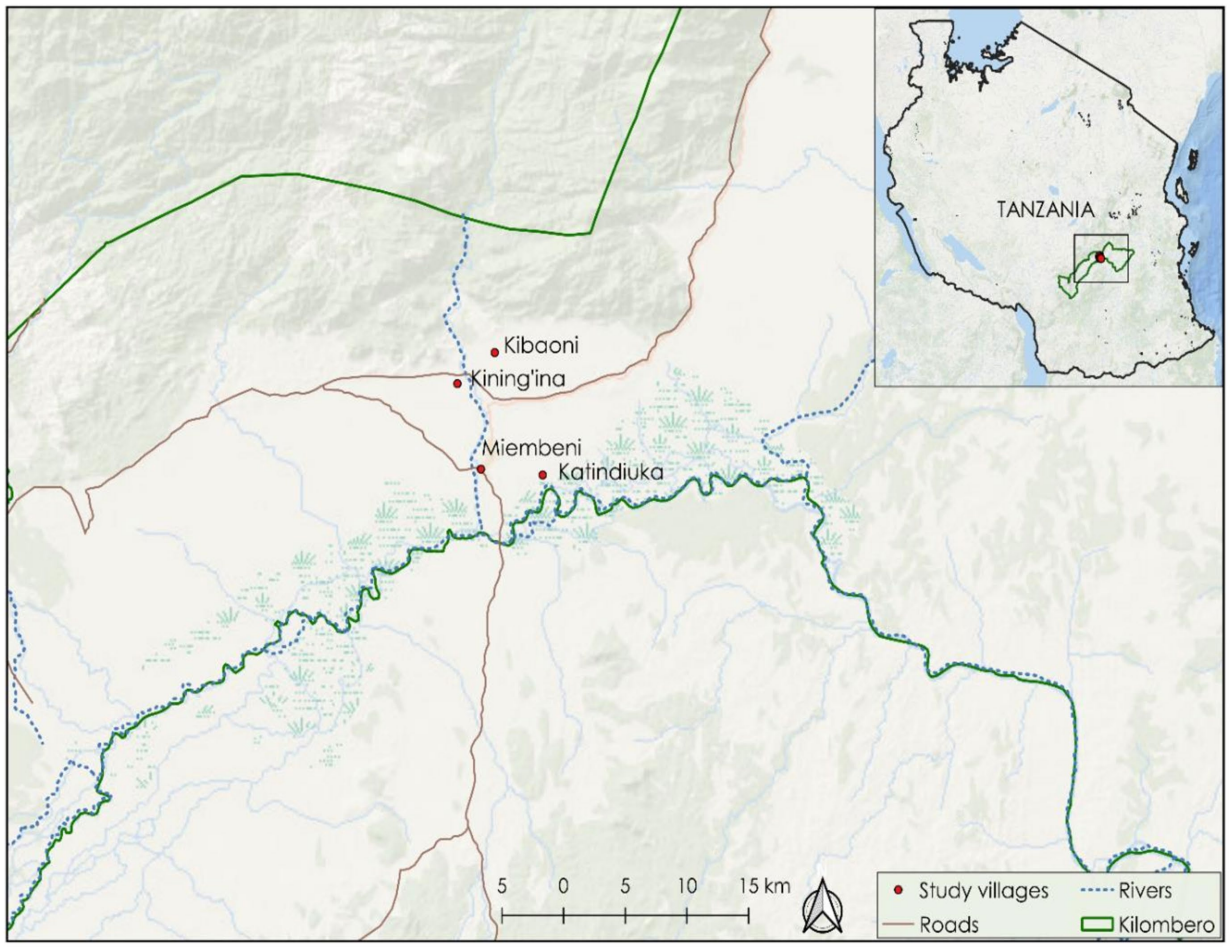
A map showing the geographic location of the schools where the KaziAfya project was implemented in the southern part of Tanzania between 2019 and 2021.

This study utilized secondary data collected through the KaziAfya study from four public primary schools in Ifakara Town Council in Southern Tanzania (58), a small but rapidly growing town in Southern Tanzania. A more detailed description of the study site is provided by Minja et al. (59, 60). The Kazi Afya project is a four-arm cluster RCT designed to assess the impact of physical activity and MMS on school children’s growth, health, and wellbeing. The KaziAfya project employed a 2 × 2 factorial design to evaluate the effects of these interventions. The schools involved in the study were Katindiuka, Kibaoni, Kining’ina, and Miembeni primary schools (Figure 1). These schools were selected through a systematic sampling process to ensure representation of different socioeconomic backgrounds within the rapidly growing town (60). The intervention aimed to promote physical activity and provide MMS. Pupils were recruited from selected classes within these schools based on criteria including age, parental consent, and baseline health status.

A total of over 900 pupils were recruited for the KaziAfya study, with 678 children providing complete baseline data on physical activity, nutritional status, and higher-order cognitive abilities. Although the current analysis is based on cross-sectional data, the original cluster RCT was designed with an a-priori power analysis to determine the sample size required to detect significant effects of the interventions (58). Following the completion of data collection, an a-posteriori power analysis was conducted using G*Power software to ensure the adequacy of the sample size in detecting associations between the primary study variables. The analysis assumed a small effect size (Cohen’s *f*^2^ = 0.02), a standard alpha level of 0.05, and a statistical power of 0.80 (*β* = 0.20), which are commonly recommended for behavioral and health research. Based on these parameters, the sample size was confirmed to be sufficiently powered to detect small yet meaningful associations. This robust methodological approach enhances the reliability and validity of the findings, ensuring that the observed results are not influenced by a lack of statistical power.

### Assessment of physical activity

Triaxial accelerometer devices (ActiGraph^®^ wGT3X-BT; Pensacola, FL, USA) were used to measure children’s physical activity levels (61). Children were given these devices to wear around their waist for seven consecutive days, only taking them off when they took a shower to avoid contact with water. The ActiGraph accelerometer recorded data 30 times per second to ensure accurate measurement of the children’s physical activity levels (62). To focus on daytime physical activity, a filter was set to extract data only between 06:00 and 24:00 h. All recordings were saved in GTX-format and analyzed using ACTi Life software version 6.13.2 (Pensacola, FL, USA) (62). For inclusion in the study, children needed valid data for at least four weekdays and at least one weekend day, with a wear time of at least 8 h per day (63). Child-specific cut-points were applied to extract minutes of MVPA per day (64).

### Assessment of nutritional status

Nutritional status was determined using a wireless body composition monitor (Tanita MC-580, Tanita Corp.; Tokyo, Japan), which measured children’s body weight (65). Height was measured using a height measuring board, and z-scores were calculated according to the WHO standards as follows: (i) less than −3 score indicated severe acute malnutrition; (ii) between −3 and −2 indicated moderate acute malnutrition; (iii) between −2 and +2 indicated normal nutritional status; (iv) between +2 and +3 indicated overweight; and (v) scores greater than +3 were classified as being obese (66).

### Assessment of higher-order cognitive abilities

Flanker task was used to understand attention, cognitive control, and reaction times toward stimuli (39, 67). The Flanker task is a computer-based test which includes both congruent and incongruent stimuli: congruent stimuli feature aligned or matching targets and surrounding distractors, whereas incongruent stimuli involve non-matching targets and surrounding distractors (46). Accuracy, in the context of cognitive tasks, refers to the proportion of correct responses made by participants. Accuracy is calculated by dividing the number of correct responses by the total number of responses, thereby providing a measure of how often participants correctly identify or respond to target stimuli amidst distractors (46, 68). In this case, computerized Flanker task was used to evaluate children’s inhibitory control and information processing, which are components of executive cognitive function (69). This task was administered using E-Prime 2.0 software from Psychology Software Tools (70). Before beginning the test, children received standardized verbal instructions from a trained researcher. The testing took place in the morning on a regular school day, with each child seated in front of a laptop in a room with a total of 10 children (58).

The stimuli presented on the screen consisted of five white fish arranged horizontally, pointing either to the right or left. The children’s task was to identify the direction of the central target fish. With one finger, children responded using the keyboard’s right and left arrows. The task lasted for about 15 min and included two practice rounds with 60 trials, followed by two rounds of 40 trials each (71). Performance metrics included accuracy (i.e., proportion of correct responses for each trial type) and mean reaction time for both congruent and incongruent trials. Performance on congruent trials was used as an indicator of information processing, while performance on incongruent trials was indicative of inhibitory control. To ensure data quality, datasets with accuracy rates lower than 50% during the second practice round were excluded from the analysis (71).

### Statistical analyses

Data were analyzed using R statistical software version 4.2.1 (72). Descriptive statistics were used to present the demographic information of the study participants. The Shapiro–Wilk’s test was used to test normality (73). A multinomial logistic regression model was used to determine the relationship between MVPA and the nutritional status of the children, whereby MVPA was used as independent (explanatory) variable and nutritional status as a dependent (response) variable. This model was implemented using *multinom()* function from *nnet* package (74). Additionally, a gamma generalized linear model (GLM) with a log link function was used to examine the association between MVPA (independent variable) and higher-order cognitive ability (dependent variable) of children, who had both valid data for accuracy and reaction time (75), specifically focusing on reaction time for incongruent stimuli as an indicator of inhibitory control. The model was adjusted for accuracy to ensure that the associations between MVPA and higher-order cognitive abilities were not confounded by varying accuracy levels. This is important because reaction times typically decrease as accuracy increases (76, 77).

### Ethical consideration

Permission to conduct this study was obtained from the Ifakara Health Institute Institutional Review Board (Ref. # IHI/IRB/No/:37–2023). Additionally, the Ifakara Health Institute (IHI), the National Institute for Medical Research (NIMR), and the Tanzania Medical Drugs Authority (TMDA) granted approval for previous studies under the KaziAfya project.

## Results

### Demographic information of the participants

A detailed summary of the sample characteristics of all children that participated in the KaziAfya study is provided by Minja et al. (59, 60). As for the 678 school children that were considered for the present data analyses, a slight majority were girls (52.2%, *n* = 354) and aged between 10 and 13 years (58.1%, *n* = 394) (Table 1). More than three-quarters (77.9%, *n* = 528) of the children had a normal weight, with the remaining children being classified as underweight (14.3%, *n* = 97), and overweight (7.8%, *n* = 53). When examining differences in nutritional status by sex and age, it was observed that among the underweight group, a higher percentage were boys (63.9%, *n* = 62) compared to girls (36.1%, *n* = 35), and more were in the age group 10–13 years (53.6%, *n* = 52).

**Table 1:** Baseline characteristics of the study participants in the KaziAfya project in Tanzania in 2019.

Variable	Category	*n* [Percentage]
Sex	Boys	324 (47.8%)
	Girls	354 (52.2%)
Age group	6–9 years	284 (41.9%)
	10–13 years	394 (58.1%)
Underweight	Boys	62 (63.9%)
	Girls	35 (36.1%)
	6–9 years	45 (46.4%)
	10–13 years	52 (53.6%)
Normal weight	Boys	247 (46.8%)
	Girls	281 (53.2%)
	6–9 years	226 (42.8%)
	10–13 years	302 (57.2%)
Overweight	Boys	15 (28.3%)
	Girls	38 (71.7%)
	6–9 years	13 (24.5%)
	10–13 years	40 (75.5%)
Meeting recommended levels of MVPA criteria	Boys	309 (49.2%)
	Girls	319 (50.8%)
	6–9 years	262 (42.0%)
	10–13 years	364 (58.0%)
Not meeting recommended levels of MVPA criteria	Boys	15 (30.0%)
	Girls	35 (70.0%)
	6–9 years	20 (40.0%)
	10–13 years	30 (60.0%)

MVPA, moderate-to-vigorous physical activity.

Similarly, within the overweight group, girls represented a significantly higher proportion (71.7%, *n* = 38) compared to boys (28.3%, *n* = 15), with the majority being in the age group 10–13 years (75.5%, *n* = 40) (Table 1).

In terms of meeting the WHO criteria for MVPA, 92.6% (*n* = 628) of the children met the recommendations (≥60 min of MVPA/day). The proportions of boys and girls meeting the MVPA criteria were nearly equal, but among those who did not meet the criteria, a higher proportion were girls (70.0%, *n* = 35) compared to boys (30.0%, *n* = 15) Regarding age, 42% (*n* = 262) of those who met the MVPA criteria were aged 6–9 years, while 58% (*n* = 364) were aged 10–13 years.

Among those who did not meet the WHO criteria, 40% (*n* = 20) were aged 6–9 years, and 60% (*n* = 30) were aged 10–13 years (Table 1).

With regards to higher-order cognitive abilities, boys showed slightly higher accuracy and faster reaction times in the Flanker task compared to girls, with boys scoring 0.88 [standard deviation (SD)  = 0.17] and 0.83 (SD = 0.22) in congruent and incongruent tasks respectively, while girls scored 0.86 (SD = 0.19) and 0.80 (SD = 0.24) (Table 2). Additionally, children aged 10–13 years had better cognitive performance than those aged 6–9 years, with higher accuracy (0.89, SD = 0.17 compared to 0.84, SD = 0.19) and faster reaction times (1080.9, SD = 259.9 compared to 1220.8, SD = 281.9 in congruent tasks and 1127.5, SD = 282.2 compared to 1272.1, SD = 303.1 in incongruent tasks) (Table 2).

**Table 2:** Sex and age variations in higher-order cognitive abilities and nutritional status of the school children participating in the KaziAfya cluster randomized controlled trial in Tanzania in 2019-2021.

		Sex difference				Age difference		
Variable	Category	All children	Boys	Girls	*p*-value	6–9 years	10–13 years	*p*-value
	Accuracy – Congruent	0.9 (0.2)	0.9 (0.2)	0.9 (0.2)	0.20	0.8 (0.2)	0.9 (0.2)	0.001
Higher-order cognitive abilities Mean (SD)	Accuracy – Incongruent	0.8 (0.2)	0.8 (0.2)	0.8 (0.2)	0.05	0.8 (0.2)	0.8 (0.2)	< 0.001
	Reaction time – Congruent*	1139.5 (277.8)	1102.8 (272.7)	1173.1 (278.6)	0.001	1220.8 (281.9)	1080.9 (259.9)	< 0.001
	Reaction time –Incongruent*	1188.1 (299.6)	1160.4 (295.5)	1112.4 (301.5)	0.021	1272.1 (303.1)	1127.5 (282.2)	< 0.001
Nutritional status *n* (%)	Normal weight	528 (77.9%)	247 (46.8%)	281 (53.2%)	0.001	226 (42.8%)	302 (57.2%)	< 0.001
	Underweight	97 (14.3%)	62 (63.9%)	35 (36.1%)	0.001	45 (46.4%)	52 (53.6%)	0.36
	Overweight/obesity	53 (7.5%)	15 (28.3%)	38 (71.7%)	0.10	13 (24.5%)	40 (75.5%)	< 0.001

*Values on reaction time are provided in millisecond. SD, standard deviation.

### Multivariate analysis of the association between physical activity and nutritional status

Children who did not meet physical activity recommendations had a higher likelihood (OR = 2.62, 95% confidence interval [CI] = 1.15–5.95, *p* = 0.022) of being overweight compared to those who met the criteria after adjusting for age and sex. Children aged 10–13 years had a significantly higher likelihood (OR = 2.41, 95% CI = 1.25–4.64, *p* = 0.009) of being overweight compared to those aged 6–9 years when adjusted to MVPA and sex. Moreover, boys had a higher likelihood (OR = 2.09, 95% CI = 1.33–3.29, *p* = 0.001) of being underweight and a lower likelihood (OR = 0.46, 95% CI = 0.24–0.85, *p* = 0.015) of being overweight than girls (Table 3).

**Table 3:** Analysis of association between physical activity and nutritional status after controlling for age and sex among school children in the KaziAfya study in Tanzania, 2019-2021.

Variable	Category	Normal weight as reference			
		Underweight		Overweight	
		OR (95% CI)	*p*-value	OR (95% CI)	*p*-value
MVPA	Met criteria	1.00		1.00	
	Did not meet criteria	1.53 (0.68–3.49)	0.305	2.62 (1.15–5.95)	0.022
Sex	Girls	1.00		1.00	
	Boys	2.09 (1.33–3.29)	0.001	0.46 (0.24–0.89)	0.015
Age	6–9 years	1.00		1.00	
	10–13 years	0.82 (0.52–1.27)	0.368	2.41 (1.25–4.64)	0.009

CI, confidence interval; MVPA, moderate-to-vigorous physical activity; OR, odds ratio.

### Association between physical activity and higher-order cognitive abilities

#### Accuracy for congruent stimuli

After adjusting for sex, children aged 10–13 years had significantly higher accuracy than those aged 6–9 years (coefficients or parameter estimates = 1.07, 95% CI: 1.03–1.10, *p* < 0.001), suggesting better performance in older children. There was no significant difference in accuracy between children meeting versus not meeting physical activity recommendations (coefficients or parameter estimates = 0.97, 95% CI: 0.91–1.03, *p* = 0.288). There was a significant (but weak) association between reaction time and accuracy for congruent stimuli (coefficients or parameter estimates = 1.00, 95% CI: 1.00–1.00, *p* = 0.021). No significant difference was observed between boys and girls (Table 4).

**Table 4:** Analysis of association between physical activity and higher-order cognitive abilities.

Cognitive performance												
	Accuracy congruent			Accuracy incongruent			Reaction time congruent			Reaction time incongruent		
Variables	Esti-mates	(95% CI)	*p*-value	Esti-mates	(95% CI)	*p*-value	Esti-mates	(95% CI)	*p*-value	Esti-mates	(95% CI)	*p*-value
Met MVPA criteria	1.00			1.00			1.00			1.00		
Did not meet MVPA criteria	0.97	0.91–1.03	0.288	0.99	(0.91–1.07)	0.81	0.98	0.92–1.05	0.557	0.98	0.91–1.05	0.525
Girls	1.00			1.00			1.00			1.00		
Boys	1.02	0.99–1.05	0.212	1.03	0.99–1.08	0.123	0.94	0.91–0.98	**0.002**	0.96	0.93–1.00	**0.048**
6–9 years	1.00			1.00			1.00			1.00		
10–13 years	1.07	1.03–1.10	**<0.001**	1.10	1.06–1.15	**<0.001**	0.88	0.85–0.92	**<0.001**	0.89	0.86–0.93	**<0.001**

Bold values indicate statistically significant results at *p* < 0.05. MVPA, moderate-to-vigorous physical activity.

#### Accuracy for incongruent stimuli

After adjusting for sex, similar to congruent tasks, older children (10–13 years) achieved significantly higher accuracy than younger peers (6–9 years) (coefficients or parameter estimates: 1.10, 95% CI: 1.06–1.15, *p* < 0.001). Physical activity criteria did not significantly affect accuracy (coefficients or parameter estimates: 1.02, 95% CI: 0.94–1.10, *p* = 0.712). Reaction time for incongruent stimuli was not significantly associated with accuracy (coefficients or parameter estimates: 1.00, 95% CI: 1.00–1.00, *p* = 0.155). A significant difference was observed between boys and girls (Table 4).

#### Reaction time for congruent stimuli

Accuracy for congruent stimuli showed a marginally significant positive association with reaction time for congruent stimuli (coefficients or parameter estimates: 1.10, 95% CI: 1.00–1.22, *p* = 0.051). Children aged 10–13 years had a significantly lower reaction time than those aged 6–9 years (coefficients or parameter estimates: 0.88, 95% CI: 0.85–0.92, *p* < 0.001), suggesting better performance in older children. There was no significant difference in reaction time for congruent stimuli between children meeting and not meeting WHO physical activity recommendations (coefficients or parameter estimates: 0.98, 95% CI: 0.92–1.05, *p* = 0.557). Boys had a slightly faster reaction time than girls (coefficients or parameter estimates 0.94, 95% CI: 0.91–0.98, *p* = 0.002) (Table 4).

#### Reaction time for incongruent stimuli

Accuracy for incongruent stimuli was not significantly associated with reaction time for incongruent stimuli (coefficients or parameter estimates: 0.94, 95% CI: 0.87–1.02, *p* = 0.166). Similar to congruent tasks, older children (10–13 years) had significantly faster reaction time compared to their younger peers (6–9 years) (coefficients or parameter estimates: 0.89, 95% CI: 0.86–0.93, *p* < 0.001). Children who met vs. did not meet physical activity recommendations did not differ in reaction time for incongruent stimuli (coefficients or parameter estimates: 0.98, 95% CI: 0.91–1.05, *p* = 0.525). Boys showed a slightly faster reaction time for incongruent stimuli than girls (coefficients or parameter estimates: 0.96, 95% CI: 0.93–1.00, *p* = 0.048) (Table 4).

#### Association between nutritional status and higher-order cognitive abilities

No significant differences were observed in reaction times for congruent and incongruent stimuli between overweight and normal weight children (*p* > 0.05). There are differences between normal weight and underweight children (*p* = 0.016), Underweight children performed faster than normal-weight children for both congruent and incongruent reaction times. No significant association was found between nutritional status and accuracy (*p* > 0.05). Older children (10–13 years) consistently exhibited faster reaction times compared to younger children (6–9 years) (*p* < 0.001). Regarding sex, boys demonstrated slightly faster reaction times than girls for stimuli (*p* = 0.009); however, this difference was not observed for incongruent stimuli (Table 5).

**Table 5:** Association between nutritional status and higher-order cognitive abilities for reaction time in children from the KaziAfya study in Tanzania in 2019-2021.

Variables	Categories	Reaction time congruent			Reaction time incongruent		
		Estimate	95% CI	*p*-value	Estimate	95% CI	*p*-value
Nutritional status	Normal	1.00					
	Underweight	0.94	0.89–0.99	**0.016**	0.93	0.88–0.98	**0.004**
	Overweight	1.04	0.98–1.12	0.227	1.02	0.95–1.09	0.645
Accuracy	Accuracy	1.10	1.00–1.22	0.057	0.94	0.87–1.02	0.160
Age group	6–9 years	1.00					
	10–13 years	0.88	0.85–0.91	**<0.001**	0.89	0.86–0.92	**<0.001**
Sex	Girls	1.00					
	Boys	0.95	0.92–0.99	**0.009**	0.97	0.94–1.01	0.129

Bold values indicate statistically significant results at *p* < 0.05.

No significant differences was observed in accuracy for underweight or overweight children compared to those with normal weight for both congruent (underweight: coefficients or parameter estimates = 0.96, 95% CI: 0.92–1.01, *p* = 0.083; overweight: coefficients or parameter estimates = 0.98, 95% CI: 0.93–1.04, *p* = 0.596) and incongruent tasks (underweight: coefficients or parameter estimates = 0.98, 95% CI: 0.92–1.04, *p* = 0.434; overweight: coefficients or parameter estimates = 0.94, 95% CI: 0.86–1.02, *p* = 0.124). Reaction time was significantly associated with accuracy for the congruent task (coefficients or parameter estimates = 1.00, 95% CI: 1.00–1.00, *p* = 0.028), but not for the incongruent task (*p* = 0.140). Older children (10–13 years) consistently achieved higher accuracy rates than younger children (6–9 years) for both congruent (coefficients or parameter estimates = 1.07, 95% CI: 1.03–1.10, *p* < 0.001) and incongruent tasks (coefficients or parameter estimates = 1.11, 95% CI: 1.06–1.16, *p* < 0.001). No significant differences in accuracy were found between boys and girls for either congruent or incongruent stimuli (*p* > 0.05) (Table 5). In addition to these results, we also conducted an additional analysis using multiple linear regression as described in Supplementary material. Importantly, the outcomes of this analysis aligned with the original findings, thereby confirming the robustness and consistency of our results.

## Discussion

To our knowledge, this is one of the first studies to examine the association between physical activity, nutritional status, and higher-order cognitive abilities in LMICs, and will therefore provide a baseline from which other studies can be conducted. In this study, more than three-quarters of the children had normal body weight, 14% were underweight, and 7% were over-weight/obese. These findings correlate with the national estimates reported by the SMNS, which indicates that 11.7% were underweight 11.0% were thin, and 5% were overweight or obese (28).

More than 90% of the children who participated in this study met the WHO recommendations for MVPA (≥60 min per day). However, these findings are in marked contrast to a previous WHO report, indicating that nearly 80% of children and adolescents in sub-Saharan Africa are physically insufficiently inactive (18). This discrepancy is likely attributable to the fact that our study sample may not be representative of the broader global population, as it included children from a rural setting who access school service by walking long distances, and who participate in physical chores at home.

Another reason might be that our findings are based on actigraphy data, whereas the WHO report relied on self-reported data. Our findings, however, are similar to other studies that have observed higher levels of physical activity in Côte d’Ivoire and South Africa, where 89.6% and 76.9% of school children met the WHO criteria for MVPA, respectively (78). These similarities in findings could be due to the similitudes in groups of children assessed and methodologies used (78).

Children who did not meet recommended levels of MVPA according to WHO criteria had nearly three times higher likelihood of being overweight compared to those who met the criteria. This is likely due to the fact that physical inactivity contributes significantly to the energy imbalance, where caloric intake exceeds caloric expenditure, leading to weight gain and, eventually, obesity (20, 21). Physical activity helps regulate energy balance by increasing energy expenditure and improving metabolic health, which can prevent excessive weight gain (20, 21). Moreover, regular physical activity has been shown to reduce the risk of obesity-related health conditions, such as insulin resistance and cardiovascular diseases, by enhancing insulin sensitivity and promoting cardiovascular fitness. Hence, children who do not engage in sufficient physical activity are at a higher risk of obesity due to both the direct effects on energy balance and the indirect effects on metabolic health (52). Similar correlations have been observed among school children in South Africa (62, 79). Similarly, a study in the United States found that children with lower levels of physical activity had significantly higher rates of obesity compared to their more active peers (80). Moreover, similar associations have been observed in Australia, showing a strong association between insufficient physical activity and increased obesity rates in children (81). In LMICs, lower physical activity levels have also been associated with higher obesity rates in Brazil, Benin, Djibouti, Egypt, Ghana, Mauritania, Malawi, and Morocco (82, 83).

In our study, boys had a lower likelihood of being obese compared to girls. This is likely due to differences in physical activity levels, hormonal influences, and socio-cultural factors that affect dietary habits and body composition (27, 84). These findings correlate with similar studies in Brazil and South Africa, where girls were observed to have higher obesity rates compared to boys, likely due to girls accumulating more body fat compared to boys (62, 82). In this study, girls were less likely to meet the MVPA criteria compared to boys, which is in line with a study done in the United States, which indicated girls to be less likely to meet the WHO criteria for MVPA (85). The older children were found to be more active compared to their younger counterparts, likely due to the fact that this age group may have developed a stronger sense of routine and discipline in maintaining an active lifestyle, possibly influenced by school programs, peer interactions, and home chores. Our findings contradict other studies that show a decline in physical activity with age, particularly among girls (80, 85). This difference is likely since children in this study were located in a rural setting, where children have different activity patterns compared to those in urban settings. On the other hand, the older children were more likely to be overweight than the younger children which is likely attributable to various factors including hormonal changes, decreased physical activity, increased sedentary behavior, and greater autonomy in food choices (86). Other similar studies have also observed similar trends of the increase in obesity among older children across Europe and the Americas (87, 88).

In contrast to our expectations, in our study, no significant association was observed between physical activity and higher-order cognitive abilities. This is in line with similar studies carried out in South Africa (71) and the southeastern part of Spain (89). This is likely due to the fact that the types and intensities of physical activities performed by the children were not sufficiently consistent or tailored to impact higher-order cognitive abilities significantly. Additionally, cognitive performance can be influenced by cultural, social, psychological, and lifestyle factors, which were not assessed in this study. However, our findings appear to contradict the conclusions drawn from several studies that have indicated that type, frequency, and intensity of exercise have an influence on cognitive performance (37, 90–93). These differences could be attributable to the different groups surveyed, and types of physical activities that were provided to the children.

In examining the association between nutritional status and higher-order cognitive abilities, our findings show that underweight children exhibit slower reaction times compared to their normal-weight peers (*p <* 0.05), while no significant differences were observed in overweight children (*p* > 0.05). The findings that underweight children exhibited slower reaction times compared to those with normal weight (*p* < 0.05) are in agreement with other studies that have shown undernutrition impairs cognitive processing speed (94, 95). This alignment may be due to the negative impact of nutrient deficiencies on brain function, particularly in areas related to attention and cognitive control (29). In contrast, no significant differences were found for overweight children (*p* > 0.05), which is consistent with studies that suggest excess weight may not directly affect cognitive performance in children (32). However, this lack of association could be influenced by confounding factors such as socioeconomic status which were not fully accounted for in this analysis (96). Similarly, the lack of significant association between nutritional status and accuracy (*p* > 0.05) aligns with findings from recent research, suggesting that while nutrition affects processing speed, it might not significantly impact accuracy in cognitive tasks (97, 98). Possible reasons for this could include task complexity and the compensatory cognitive strategies employed by children. This supports existing evidence that malnutrition can impact cognitive processing speed, though its effect on accuracy appears limited, as neither underweight nor overweight children showed significant differences in accuracy compared to normal-weight peers (*p* > 0.05). This finding aligns with similar studies done in South Africa under the same project called KaziAfya with same methodologies (99). It also aligns with others studies in LMICs (100–102). Furthermore, recent research in rural Tanzania also supports the link between cognitive function and nutritional status. This study examined psychomotor speed and ocular movements in relation to nutritional status, providing valuable insights that complement our findings. While both studies show the importance of nutrition in cognitive performance, the types of cognitive tests and physical activity assessments used differ. Our study contributes by focusing on physical activity levels, whereas the other study emphasizes psychomotor speed and eye tracking, highlighting the diverse methods and outcomes in this field (1).

Moreover, older children (10–13 years) demonstrated improved cognitive performance in reaction time and accuracy tasks relative to younger children (6–9 years) (*p* < 0.001), in line with developmental theories indicting cognitive gains with age (103).

Gender differences were minimal, with boys showing slightly faster reaction times than girls, but no significant accuracy differences, which aligns with findings from different existing theories (104). The findings underscore that while undernutrition may impair certain cognitive functions, the effect on task accuracy remains inconclusive (105). The finding that older children (10–13 years) performed better in both reaction time and accuracy aligns with developmental theories, indicating that cognitive functions like inhibitory control improve with age (106). The slight gender differences observed in reaction time, but not in accuracy, which align with studies that report no difference based on sex in cognitive function (107, 108).

### Strength and limitation of the study

A key strength of this study is the robust methodological approach, including the use of a triaxial accelerometer for objective physical activity measurement, a Flanker task for cognitive assessment, and high-precision equipment for anthropometric measurements. Additionally, the study highlights that a significant proportion of children (92.6%) met the recommended physical activity levels, aligning with global standards.

However, certain limitations must be acknowledged. While the Flanker task provides valuable insights into cognitive performance, the unfamiliarity of the target population with computerized tasks may have influenced their performance. Furthermore, as a cross-sectional study, this research cannot establish causal relationships, but instead only derive associations between the variables analyzed. The findings did not indicate significant associations between physical activity and cognitive performance or between nutritional status and cognitive performance, which presents a challenge in drawing direct conclusions about these relationships. Lastly, since the study was conducted in a specific rural context, the results may not be generalizable to urban populations or different geographical regions. However, despite these limitations, the foundational nature of our findings remains significant. Indeed, our findings provide valuable insights into the dynamics and benefits of physical activity among children in rural African settings and serve as a foundational reference for future, more comprehensive studies.

## Conclusion

This study highlights the relevance of promoting physical activity in preventing childhood obesity in sub-Saharan Africa. Children who failed to meet the WHO criteria for physical activity faced a significantly higher risk of being classified as overweight/obese, highlighting the necessity for interventions that encourage participation in regular physical activity. Despite not finding a significant association between physical activity and higher-order cognitive abilities in our study, further research is essential to explore this relationship in more detail. Understanding the factors that impact children’s physical and cognitive development is crucial for designing effective interventions that promote overall health and wellbeing.

## Data Availability

The raw data supporting the conclusions of this article will be made available by the authors, without undue reservation.

## References

[1] Strain T Flaxman S Guthold R Semenova E Cowan M Riley LM. National, regional, and global trends in insufficient physical activity among adults from 2000 to 2022: a pooled analysis of 507 population-based surveys with 5·7 million participants. Lancet Glob Heal. (2024) 12:e1232–43. doi: 10.1016/S2214-109X(24)00150-5, PMID: 38942042 PMC11254784

[2] Early W Obesity C Initiative P Start H Taken S Activity P. Physical activity guidelines for Americans. Kittyhawk Digital (2013). 56–63.

[3] Di Liegro CM Schiera G Proia P Di Liegro I. Physical activity and brain health. Genes. (2019) 10:720. doi: 10.3390/genes10090720, PMID: 31533339 PMC6770965

[4] Erickson KI Hillman C Stillman CM Ballard RM Bloodgood B Conroy DE. Physical activity, cognition, and brain outcomes: a review of the 2018 physical activity guidelines. Med Sci Sports Exerc. (2019) 51:1242–51. doi: 10.1249/MSS.0000000000001936, PMID: 31095081 PMC6527141

[5] Swift DL Johannsen NM Lavie CJ Earnest CP Church TS. The role of exercise and physical activity in weight loss and maintenance. Prog Cardiovasc Dis. (2014) 56:441–7. doi: 10.1016/j.pcad.2013.09.012, PMID: 24438736 PMC3925973

[6] Jakicic JM Powell KE Campbell WW Dipietro L Pate RR Pescatello LS. Physical activity and the prevention of weight gain in adults: a systematic review. Med Sci Sports Exerc. (2019) 51:1262–9. doi: 10.1249/MSS.0000000000001938, PMID: 31095083 PMC6527311

[7] Elagizi A. A review of obesity, physical activity, and cardiovascular disease. Curr Obes Rep. (2020) 9:571–81. doi: 10.1007/s13679-020-00403-z, PMID: 32870465

[8] Chalkley A. A rapid evidence review muscle and bone strengthening activities for children and young people (5 to 18 Years): a rapid evidence review 2 about public health England. London, UK: Public Health England (2021).

[9] Weaver CM Gordon CM Janz KF Kalkwarf HJ Lappe JM Lewis R. The National Osteoporosis Foundation’s position statement on peak bone mass development and lifestyle factors: a systematic review and implementation recommendations. Osteoporos Int. (2016) 27:1281–386. doi: 10.1007/s00198-015-3440-3, PMID: 26856587 PMC4791473

[10] Syed A. The important of physical activities in our life. Int Phys Med Rehabil J. (2018) 3:308–10. doi: 10.15406/ipmrj.2018.03.00121, PMID: 40314456

[11] Marmeleira J Ferreira S Raimundo A. Physical activity and physical fitness of nursing home residents with cognitive impairment: a pilot study. Exp Gerontol. (2017) 100:63–9. doi: 10.1016/j.exger.2017.10.025, PMID: 29107061

[12] WHO. Promoting physical activity through school: policy brief. Geneva: World Health Organization (2022).

[13] Patrício DS Hilgemberg GR Marques LHSC Maceno MBS Matias JMF Carvalho LPC. Physical activities and contributions to the prevention of chronic non-communicable diseases. Multidiscip Perspect Integr Knowl. (2024). doi: 10.56238/sevened2024.007-087

[14] Lopez-Bueno R Bláfoss R Calatayud J Lopez-Sanchez GF Smith L Andersen LL. Association between physical activity and odds of chronic conditions among workers in Spain. Prev Chronic Dis. (2020) 17:1–13. doi: 10.5888/pcd17.200105, PMID: 33034558 PMC7553219

[15] DiPietro L Buchner DM Marquez DX Pate RR Pescatello LS Whitt-Glover MC. New scientific basis for the 2018 U.S. Phys Act Guidelines J Sport Heal Sci. (2019) 8:197–200. doi: 10.1016/j.jshs.2019.03.007, PMID: 31193291 PMC6525104

[16] Mzumara T Banda O. Physical activity and its relationship with diabetes. Hypertension Soc Demogr Fact Rural. (2024):1–17. doi: 10.21203/rs.3.rs-4076135/v1

[17] Okely AD Kontsevaya A Ng J Abdeta C. 2020 WHO guidelines on physical activity and sedentary behavior. Sport Med Heal Sci. (2021) 3:115–8. doi: 10.1016/j.smhs.2021.05.001, PMID: 35782159 PMC9219310

[18] World Health Organization. Global status report on physical activity 2022. Geneva: World Health Organization. (2022). p. 1–112

[19] World Health Organization. Physical-activity-tza-2022-country-profile. World Health Organization (2022).

[20] Huang Q Zhao J Jiang W Wang W. The association between physical activity and cognitive function: data from the China Health and Nutrition Survey. Behav Neurol. (2022) 2022:1–12. doi: 10.1155/2022/3438078 PMC9236826 35769517

[21] Munawar YG Lontoh SO. The association between physical activity with the nutritional status of student in Faculty of Medicine Tarumanagara University in 2019-2020. Adv Health Sci Res. (2021) 41:6–10.

[22] Reber E Gomes F Vasiloglou MF Schuetz P Stanga Z. Nutritional risk screening and assessment. J Clin Med. (2019) 8:1–19. doi: 10.3390/jcm8071065, PMID: 31330781 PMC6679209

[23] WHO. Nutritional status amoung different age group. World Health Organization (2024).

[24] Sanyaolu A Okorie C Qi X Locke J Rehman S. Childhood and adolescent obesity in the United States: a public health concern. Glob Pediatr Heal. (2019) 6:6. doi: 10.1177/2333794X19891305, PMID: 31832491 PMC6887808

[25] Khan DSA Das JK Zareen S Lassi ZS Salman A Raashid M. Nutritional status and dietary intake of school-age children and early adolescents: systematic review in a developing country and lessons for the global perspective. Front Nutr. (2022) 8:739447. doi: 10.3389/fnut.2021.739447, PMID: 35187014 PMC8848764

[26] Mekonnen T Tariku A Abebe SM. Overweight/obesity among school aged children in Bahir Dar City: cross sectional study. Ital J Pediatr. (2018) 44:1–8. doi: 10.1186/s13052-018-0452-6, PMID: 29361952 PMC5781282

[27] Amoadu M Abraham SA Adams AK Akoto-Buabeng W Obeng P Hagan JE. Risk factors of malnutrition among in-school children and adolescents in developing countries: a scoping review. Children. (2024) 11:01–19. doi: 10.3390/children11040476, PMID: 38671693 PMC11049343

[28] Ministry of Health Tanzania M. The 2021 school malaria and nutrition survey (SMNS) report i United Republic of Tanzania Ministry of Health National Malaria Control Programme. The 2021 school malaria and nutrition survey (SMNS) report the 2021 school malaria and nutrition survey (SMNS)R. United Republic of Tanzania: National Malaria Control Program (2022).

[29] Prado EL Dewey KG. Nutrition and brain development in early life. Nutr Rev. (2014) 72:267–84. doi: 10.1111/nure.12102 24684384

[30] Georgieff MK Ramel SE Cusick SE. Nutritional influences on brain development. Acta Paediatr Int J Paediatr. (2018) 107:1310–21. doi: 10.1111/apa.14287, PMID: 29468731 PMC6045434

[31] Marinoni M Giordani E Mosconi C Rosolen V Concina F Fiori F. Are dietary patterns related to cognitive performance in 7-year-old children? Evidence from a birth cohort in Friuli Venezia Giulia, Italy. Nutrients. (2022) 14. doi: 10.3390/nu14194168, PMID: 36235820 PMC9571625

[32] Nguyen JCD Killcross AS Jenkins TA. Obesity and cognitive decline: role of inflammation and vascular changes. Front Neurosci. (2014) 8:1–9. doi: 10.3389/fnins.2014.00375, PMID: 25477778 PMC4237034

[33] Zhang Q Jin K Chen B Liu R Cheng S Zhang Y. Overnutrition induced cognitive impairment: insulin resistance, gut-brain axis, and neuroinflammation. Front Neurosci. (2022) 16:884579. doi: 10.3389/fnins.2022.884579, PMID: 35873818 PMC9298971

[34] Fadó R Molins A Rojas R Casals N. Feeding the brain: effect of nutrients on cognition, synaptic function, and AMPA receptors. Nutrients. (2022) 14:1–36. doi: 10.3390/nu14194137, PMID: 36235789 PMC9572450

[35] Mou Y Blok E Barroso M Jansen PW White T Voortman T. Dietary patterns, brain morphology and cognitive performance in children: results from a prospective population-based study. Eur J Epidemiol. (2023) 38:669–87. doi: 10.1007/s10654-023-01012-5, PMID: 37155025 PMC10232626

[36] Klussman K Langer J Nichols AL. The relationship between physical activity, health, and well-being: type of exercise and self-connection as moderators. Eur J Heal Psychol. (2021) 28:59–70. doi: 10.1027/2512-8442/a000070

[37] Palareti G Legnani C Cosmi B Antonucci E Erba N Poli D. Comparison between different D-dimer cutoff values to assess the individual risk of recurrent venous thromboembolism: analysis of results obtained in the DULCIS study. Int J Lab Hematol. (2016) 38:42–9. doi: 10.1111/ijlh.12426, PMID: 26362346

[38] Salehinejad MA Ghanavati E Rashid MHA Nitsche MA. Hot and cold executive functions in the brain: a prefrontal-cingular network. Brain Neurosci Adv. (2021) 5:239821282110077. doi: 10.1177/23982128211007769, PMID: 33997292 PMC8076773

[39] Stroop JR. Studies of interference in serial verbal reactions. J Exp Psychol. (1935) 18:643–62. doi: 10.1037/h0054651

[40] Diamond A. Executive functions. Handb Clin Neurol. (2020) 173:225–40. doi: 10.1016/B978-0-444-64150-2.00020-4, PMID: 32958176

[41] Diamond A. Executive functions. Annu Rev Psychol. (2013) 64:135–68. doi: 10.1146/annurev-psych-113011-143750, PMID: 23020641 PMC4084861

[42] Walubita G Matafwali B Chansa-Kabali T Mwanza-Kabaghe S Chongwe G Kasonde-Ng’andu S. Sex and school differences in executive function performance of Zambian public preschoolers. Am J Appl Psychol. (2022) 10:31–9. doi: 10.12691/ajap-10-1-5

[43] Moffitt TE Arseneault L Belsky D Dickson N Hancox RJ Harrington HL. A gradient of childhood self-control predicts health, wealth, and public safety. Proc Natl Acad Sci USA. (2011) 108:2693–8. doi: 10.1073/pnas.1010076108, PMID: 21262822 PMC3041102

[44] Duckworth A Steinberg L. Unpacking self-control understanding and cultivating self-control in children. Child Dev Perspect. (2015) 9:32–7. doi: 10.1111/cdep.12107, PMID: 25821515 PMC4372146

[45] Silva E Sá AA. Educational challenges in the Portuguese UNESCO global geoparks: contributing for the implementation of the SDG 4. Int J Geoheritage Park. (2018) 6:95–106. doi: 10.17149/ijg.j.issn.2210.3382.2018.01.007

[46] Lamb DG Porges ES. Flanker task. Encycl Clin Neuropsychol. (2018) 16:1444–5.

[47] Hasdiana U. WHO health statistic on SDGs. Anal Biochem. (2018) 11:1–5.

[48] Center for Disease Control and Prevention. Physical activity facts. Physical Activity Facts CDC Heal Sch. (2016):1–2.

[49] Bangsbo J Krustrup P Duda J Hillman C Andersen LB Weiss M. The Copenhagen consensus conference 2016: children, youth, and physical activity in schools and during leisure time. Br J Sports Med. (2016) 50:1177–8. doi: 10.1136/bjsports-2016-096325, PMID: 27354718 PMC5036221

[50] Lima RA Larsen LR Bugge A Andersen LB. Physical fitness is longitudinally associated with academic performance during childhood and adolescence, and waist circumference mediated the relationship. Pediatr Exerc Sci. (2018) 30:317–25. doi: 10.1123/pes.2017-0206, PMID: 29526135

[51] Castelli DM Centeio EE Hwang J Barcelona JM Glowacki EM Calvert HG. The history of physical activity and academic performance research: informing the future source: monographs of the Society for Research in Child Development. The Relation of Childhood Physical Activity to Brain Health, Cogni. (2014) 79:119–48. doi: 10.1111/mono.12133, PMID: 25387418

[52] Janssen I LeBlanc AG. Systematic review of the health benefits of physical activity and fitness in school-aged children and youth. Int J Behav Nutrition and Physical Activity. (2010) 7:1–16. doi: 10.1186/1479-5868-7-40 PMC2885312 20459784

[53] Jiménez-Pav́n D Kelly J Reilly JJ. Associations between objectively measured habitual physical activity and adiposity in children and adolescents: systematic review. Int J Pediatr Obes. (2010) 5:3–18. doi: 10.3109/17477160903067601, PMID: 19562608

[54] Miguel-Berges ML Jiménez-Pavón D Moreno LA. Associations between pedometer- determined physical activity and adiposity in children and adolescents: systematic review. Rev Andaluza Med del Deport. (2015) 8:35. doi: 10.1016/j.ramd.2014.10.039 28704256

[55] Ring-Dimitriou S Krustrup P Coelho-E-Silva MJ Mota J Seabra A Rego C. Could sport be part of pediatric obesity prevention and treatment? Expert conclusions from the 28th European Childhood Obesity Group Congress. J Sport Heal Sci. (2019) 8:350–2. doi: 10.1016/j.jshs.2019.01.007, PMID: 31333888 PMC6620416

[56] Ørntoft C Fuller CW Larsen MN Bangsbo J Dvorak J Krustrup P. “FIFA 11 for health” for Europe. II: effect on health markers and physical fitness in Danish schoolchildren aged 10-12 years. Br J Sports Med. (2016) 50:1394–9. doi: 10.1136/bjsports-2016-096124 27130927 PMC5136709

[57] Chen P Wang D Shen H Yu L Gao Q Mao L. Physical activity and health in Chinese children and adolescents: expert consensus statement (2020). Br J Sports Med. (2020) 54:1321–31. doi: 10.1136/bjsports-2020-102261, PMID: 32471813 PMC7606574

[58] Gerber M Ayekoé SA Beckmann J Bonfoh B Coulibaly JT Daouda D. Effects of school-based physical activity and multi-micronutrient supplementation intervention on growth, health and well-being of schoolchildren in three African countries: the KaziAfya cluster randomised controlled trial protocol with a 2 × 2 factorial. Trials. (2020) 21:22. doi: 10.1186/s13063-019-3883-5, PMID: 31907019 PMC6945709

[59] Minja EG Swai JK Mponzi W Ngowo H Okumu F Gerber M. Dietary diversity among households living in Kilombero district, in Morogoro region, south-eastern Tanzania. J Agric Food Res. (2021) 5:100171. doi: 10.1016/j.jafr.2021.100171, PMID: 40312253

[60] Minja EG Mrimi EC Mponzi WP Mollel GJ Lang C Beckmann J. Prevalence and determinants of undernutrition in schoolchildren in the Kilombero District, south-eastern Tanzania. Trop Med Infect Dis. (2024) 9:96. doi: 10.3390/tropicalmed9050096, PMID: 38787029 PMC11125975

[61] Trost SG O’Neil M. Clinical use of objective measures of physical activity. Br J Sports Med. (2014) 48:178–81. doi: 10.1136/bjsports-2013-093173, PMID: 24311601

[62] Gerber M Lang C Beckmann J du Randt R Long KZ Müller I. Physical activity, sedentary behaviour, weight status, and body composition among South African primary schoolchildren. Int J Environ Res Public Health. (2022) 19:11836. doi: 10.3390/ijerph191811836, PMID: 36142108 PMC9517541

[63] Aadland E Andersen LB Skrede T Ekelund U Anderssen SA Resaland GK. Reproducibility of objectively measured physical activity and sedentary time over two seasons in children; comparing a day-by-day and a week-by-week approach. PLoS One. (2017) 12:e0189304. doi: 10.1371/journal.pone.0189304 PMC5720738 29216318

[64] Evenson KR Catellier DJ Gill K Ondrak KS McMurray RG. Calibration of two objective measures of physical activity for children. J Sports Sci. (2008) 26:1557–65. doi: 10.1080/02640410802334196, PMID: 18949660

[65] Long KZ Beckmann J Lang C Seelig H Nqweniso S Probst-Hensch N. Associations of growth impairment and body composition among south african school- aged children enrolled in the KaziAfya project. Nutrients. (2021) 13:2735. doi: 10.3390/nu13082735, PMID: 34444895 PMC8399056

[66] Chokroverty L. Children and adolescents. A Guid to Glob Ment Heal Pract Seeing Unseen. (2015) 2015:93–110. doi: 10.4324/9781315777221-9, PMID: 40143995

[67] Botvinick MM Braver TS Barch DM Carter CS Cohen JD. Conflict monitoring and cognitive control. Psychol Rev. (2001) 108:624–52. doi: 10.1037/0033-295X.108.3.624, PMID: 11488380

[68] Fan J McCandliss BD Fossella J Flombaum JI Posner MI. The activation of attentional networks. Neuroimage. (2005) 26:471–9. doi: 10.1016/j.neuroimage.2005.02.004, PMID: 15907304

[69] Rueda MR Posner MI Rothbart MK. The development of executive attention: contributions to the emergence of self-regulation. Dev Neuropsychol. (2005) 28:573–94. doi: 10.1207/s15326942dn2802_2, PMID: 16144428

[70] Schneider W Eschman A Zuccolotto A. E-Prime 2.0 Reference Guide Manual. Psychol Softw Tools, Inc. (2012)

[71] Beckmann J Nqweniso S Ludyga S due Randt R Gresse A Long KZ. Evaluation of a physical activity and multi-micronutrient intervention on cognitive and academic performance in South African primary schoolchildren. Nutrients. (2022) 14:2609. doi: 10.3390/nu14132609, PMID: 35807790 PMC9268611

[72] R Core Team. R: A language and environment for statistical computing. R: A language and 775 environments for statistical computing. Austria: R Foundation for Statistical Computing (2016).

[73] González-Estrada E Villaseñor JA Acosta-Pech R. Shapiro-Wilk test for multivariate skew-normality. Comput Stat. (2022) 37:1985–2001. doi: 10.1007/s00180-021-01188-y

[74] Ripley B Venables W, (Maintainer). Package “nnet” needs compilation yes. Basel, Switzerland: MDPI (2023).

[75] Winter B. Generalized linear models 1. In: Statistics for linguists: an introduction using R. Imprint Routledge, p. 20.

[76] Kyllonen PC Zu J. Use of response time for measuring cognitive ability. J Intelligence. (2016) 4:1–29. doi: 10.3390/jintelligence4040014

[77] Lubczyk T Lukács G Ansorge U. Speed versus accuracy instructions in the response time concealed information test. Cogn Res Princ Implic. (2022) 7. doi: 10.1186/s41235-021-00352-8, PMID: 35006396 PMC8748592

[78] Gerber M Ayekoé SA Beckmann J Bonfoh B Kouassi KB Gba BC. Moderate-to- vigorous physical activity is associated with cardiorespiratory fitness among primary schoolchildren living in Côte d’Ivoire, South Africa, and Tanzania. Front Public Heal. (2021) 9:671782. doi: 10.3389/fpubh.2021.671782, PMID: 34490179 PMC8416979

[79] Micklesfield LK Pedro TM Kahn K Kinsman J Pettifor JM Tollman S. Physical activity and sedentary behavior among adolescents in rural South Africa: levels, patterns and correlates. BMC Public Health. (2014) 14:40. doi: 10.1186/1471-2458-14-40, PMID: 24433276 PMC3897951

[80] Tremblay MS Barnes JD González SA Katzmarzyk PT Onywera VO Reilly JJ. Global matrix 2.0: report card grades on the physical activity of children and youth comparing 38 countries. J Phys Act Health. (2016) 13:S343–66. doi: 10.1123/jpah.2016-0594 27848745

[81] Hesketh K Wake M Waters E. Body mass index and parent-reported self-esteem in elementary school children: evidence for a causal relationship. Int J Obes. (2004) 28:1233–7. doi: 10.1038/sj.ijo.0802624, PMID: 15314637

[82] Crespo NC Corder K Marshall S Norman GJ Patrick K Sallis JF. An examination of multilevel factors that may explain gender differences in children’s physical activity. J Phys Act Health. (2013) 10:982–92. doi: 10.1123/jpah.10.7.982, PMID: 23132842

[83] Manyanga T El-Sayed H Doku DT Randall JR Manyanga T El-Sayed H. The prevalence of underweight, overweight, obesity and associated risk factors among school-going adolescents in seven African countries. BMC Public Health. (2014) 14:887. doi: 10.1186/1471-2458-14-887 25168589 PMC4158085

[84] Wells JCK. Sexual dimorphism of body composition. Best Pract Res Clin Endocrinol Metab. (2007) 21:415–30. doi: 10.1016/j.beem.2007.04.007, PMID: 17875489

[85] Hallal PC Andersen LB. Physical activity levels of the world’s population. Lancet. (2012) 380:247–57. doi: 10.1016/S0140-6736(12)60646-1, PMID: 22818937

[86] Clemente E Cabral MD Senti M Patel DR. Challenges in the management of obesity in adolescents: an American perspective: a narrative review. Pediatr Med. (2022) 5:5. doi: 10.21037/pm-21-23, PMID: 40256487

[87] Kumar S Kelly AS. Review of childhood obesity: from epidemiology, etiology, and comorbidities to clinical assessment and treatment. Mayo Clin Proc. (2017) 92:251–65. doi: 10.1016/j.mayocp.2016.09.017 28065514

[88] Simmonds M Llewellyn A Owen CG Woolacott N. Predicting adult obesity from childhood obesity: a systematic review and meta-analysis. Obes Rev. (2016) 17:95–107. doi: 10.1111/obr.12334 26696565

[89] Ardoy DN Fernández-Rodríguez JM Jiménez-Pavón D Castillo R Ruiz JR Ortega FB. A physical education trial improves adolescents’ cognitive performance and academic achievement: the EDUFIT study. Scand J Med Sci Sport. (2014) 24e52-61. doi: 10.1111/sms.12093, PMID: 23826633

[90] Ludyga S Gerber M Pühse U Looser VN Kamijo K. Systematic review and meta- analysis investigating moderators of long-term effects of exercise on cognition in healthy individuals. Nat Hum Behav. (2020) 4:603–12. doi: 10.1038/s41562-020-0851-8, PMID: 32231280

[91] Greco G De Ronzi R. Effect of karate training on social, emotional, and executive functioning in children with autism spectrum disorder. J Phys Educ Sport. (2020) 20:1637–45.

[92] Szabo-Reed AN Hillman CH Greene JL Hansen DM Gibson CA Sullivan DK. Physical activity and academic achievement across the curriculum: results from a 3-year cluster-randomized trial. Prev Med. (2017) 99:140–5. doi: 10.1016/j.ypmed.2017.02.006 28193490 PMC6148354

[93] Pan CY Chu CH Tsai CL Lo SY Cheng YW Liu YJ. A racket-sport intervention improves behavioral and cognitive performance in children with attention- deficit/hyperactivity disorder. Res Dev Disabil. (2016) 57:1–10. doi: 10.1016/j.ridd.2016.06.009, PMID: 27344348

[94] Sandjaja S Poh BK Rojroonwasinkul N Le Nyugen BK Budiman B Ng LO. Relationship between anthropometric indicators and cognitive performance in Southeast Asian school-aged children. Br J Nutr. (2013) 110:S57–64. doi: 10.1017/S0007114513002079, PMID: 24016767

[95] Suryawan A Jalaludin MY Poh BK Sanusi R Tan VMH Geurts JM. Malnutrition in early life and its neurodevelopmental and cognitive consequences: a scoping review. Nutr Res Rev. (2022) 35:136–49. doi: 10.1017/S0954422421000159 34100353

[96] Sanmarchi F Scheier LM Dallolio L Ricci M Longo G Ceciliani A. Association of socioeconomic factors and physical activity with health-related quality of life in Italian middle school children: an exploratory cross-sectional study. Healthc. (2023) 11:1–19. doi: 10.3390/healthcare11142092, PMID: 37510533 PMC10379006

[97] R Cardoso B Machado P Steele EM. Association between ultra-processed food consumption and cognitive performance in US older adults: a cross-sectional analysis of the NHANES 2011–2014. Eur J Nutr. (2022) 61:3975–85. doi: 10.1007/s00394-022-02911-1, PMID: 35778619 PMC9596521

[98] Rotondi S Tartaglione L Pasquali M Ceravolo MJ Mitterhofer AP Noce A. Association between cognitive impairment and malnutrition in hemodialysis patients: two sides of the same coin. Nutrients. (2023) 15:813. doi: 10.3390/nu15040813, PMID: 36839171 PMC9964006

[99] Beckmann J Lang C du Randt R Gresse A Long KZ Ludyga S. Prevalence of stunting and relationship between stunting and associated risk factors with academic achievement and cognitive function: a cross-sectional study with South African primary school children. Int J Environ Res Public Health. (2021) 18:4218. doi: 10.3390/ijerph18084218 PMC8072858 33923436

[100] Ansuya NBS Unnikrishnan B Shashidhara YN Mundkur SC. Effect of nutrition intervention on cognitive development among malnourished preschool children: randomized controlled trial. Sci Rep. (2023) 13:10636. doi: 10.1038/s41598-023-36841-7, PMID: 37391472 PMC10313707

[101] Leroy JL Ruel M Habicht JP Frongillo EA. Linear growth deficit continues to accumulate beyond the first 1000 days in low- and middle-income countries: global evidence from 51 national surveys. J Nutr. (2014) 144:1460–6. doi: 10.3945/jn.114.191981 24944283

[102] Kar BR Rao SL Chandramouli BA. Cognitive development in children with chronic protein energy malnutrition. Behav Brain Funct. (2008) 4:31 doi: 10.1186/1744-9081-4-31, PMID: 18652660 PMC2519065

[103] Deary IJ Der G. Reaction time, age, and cognitive ability: longitudinal findings from age 16 to 63 years in representative population samples. Aging Neuropsychol Cogn. (2005) 12:187–215. doi: 10.1080/13825580590969235

[104] Leaper C. Gender and social-cognitive development. In: LS Liben, U Müller, RM Lerner, editors. *Handbook of child psychology and developmental science: cognitive processes (7th ed)*. John Wiley & Sons Inc. (2015) p. 806–853.

[105] Roberts M Tolar-Peterson T Reynolds A Wall C Reeder N Rico MG. The effects of nutritional interventions on the cognitive development of preschool-age children: a systematic review. Nutrients. (2022) 14:532. doi: 10.3390/nu14030532, PMID: 35276891 PMC8839299

[106] Steinberg J Barris R. Psychiatry and neuroscience. Curr Psychiatr Ther. (2019) 18:1–2.

[107] Alarcón G Cservenka A Fair DA Nagel BJ. Sex differences in the neural substrates of spatial working memory during adolescence are not mediated by endogenous testosterone. Brain Res. (2014) 1593:40–54. doi: 10.1016/j.brainres.2014.09.057, PMID: 25312831 PMC4252582

[108] Szadvári I Ostatníková D Babková Durdiaková J. Sex differences matter: males and females are equal but not the same. Physiol Behav. (2023) 259:114038. doi: 10.1016/j.physbeh.2022.114038, PMID: 36423797

