# Supplementary material for: Association between physical activity, nutritional status and cognitive performance among school children in southern Tanzania
Source: Front Public Health. 2025 Jun 10;13:1552215. doi: 10.3389/fpubh.2025.1552215 (PMC12185417; doi:10.3389/fpubh.2025.1552215)
Supplement: Supplementary file 1 [file Data_Sheet_1.pdf]

Supplement: Sensitivity analysis with continuous variable

Multivariate analysis predicting nutritional status (zBMI) with age, sex and physical activity
In response to the Editor-in-Chief's request and a comment of one of the anonymous reviewers, a sensitivity analysis was conducted using zBMI as a continuous outcome variable. This was done to verify the robustness of the original findings, which used categorized zBMI and multinomial logistic regression.

Table 1. Multiple linear regression predicting zBMI as a continuous outcome

Predictors	Coefficients	CI	*p*
Age (continuous)	0.126	0.069 – 0.184	**<0.001**
MVPA (continuous)	-0.001	-0.004 – 0.002	0.681
Female	Ref		
Male	-0.487	-0.681 – -0.294	**<0.001**

In this multiple linear regression model (Table 1), we assessed the impact of key predictors on children's zBMI using a multiple linear regression model, treating zBMI as a continuous outcome. The results show a statistically significant positive association between **age** and zBMI ($\beta = 0.126$, 95% CI: 0.069 to 0.184, $p < 0.001$), suggesting that as children get older, their zBMI tends to increase, with each additional year of age increasing zBMI by 0.126 (95% CI: 0.069 to 0.184, $p < 0.001$), suggesting that as children grow older, their body mass tends to increase, indicating a natural progression toward higher body mass over time. This aligns with expectations of increasing adiposity as children grow. **Sex** also showed a significant association with zBMI, with male children exhibiting lower zBMI scores than female children ($\beta = -0.487$, 95% CI: -0.681 to -0.294, $p < 0.001$). This sex difference is consistent with our primary analysis using categorized zBMI, where boys were more likely to be underweight compared to girls, reinforcing the robustness of this finding across different statistical approaches. In contrast, **MVPA (moderate-to-vigorous physical activity)** did not show a significant linear relationship with zBMI ($\beta = -0.001$, 95% CI: -0.004 to 0.002, $p = 0.681$), suggesting that while MVPA may influence nutritional status categorically (e.g., overweight/underweight), its continuous impact on zBMI across the full spectrum is negligible. This discrepancy may be attributed to the fact that the effect of MVPA on zBMI could be non-linear or threshold-dependent, which is better captured when zBMI is treated categorically. These findings support the robustness of the initial analysis, reinforcing the importance of age and sex in determining children's nutritional status. This result is also shown by the forest plot below (Figure 1).

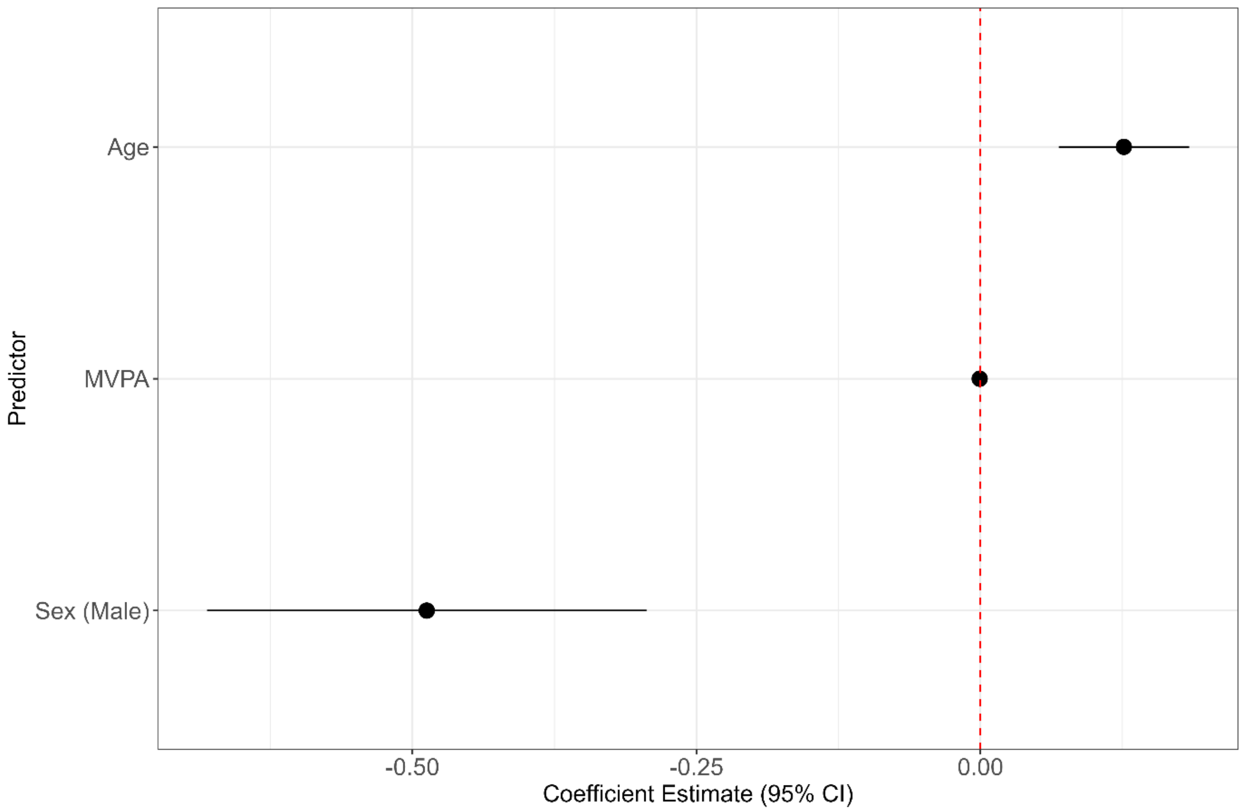

Figure 1. Forest plot showing analysis predicting nutritional status (zBMI) with age, sex and physical activity

Multivariate analysis predicting higher-order cognitive abilities with age, sex, physical activity and nutritional status

In these additional multiple linear regression analyses, we examined the predictors of higher-order cognitive abilities using **age**, **zBMI**, and **MVPA** as continuous variables. The results show that **age** consistently predicted **better cognitive performance**, with older children demonstrating both **higher accuracy** (Tables 2 & 3) and **faster reaction times** (Tables 4 & 5) across conditions (congruent vs. incongruent) (p < 0.001). This finding is entirely consistent with the original categorical analysis in the main manuscript, where children aged 10–13 outperformed those aged 6–9 years. **zBMI** was not associated with accuracy in either condition, which aligns well with our original results presented in the main manuscript where **no significant differences in accuracy** were observed across nutritional status categories. However, **higher zBMI was significantly associated with slower reaction times** in both congruent and incongruent tasks (Tables 4 & 5), complementing the original analysis which found that **underweight children responded faster than normal-weight peers**. The new continuous analysis confirms a linear association between body mass and slower cognitive speed. In contrast, **MVPA** was not a significant predictor of either accuracy or reaction time in any model, aligning with the original findings presented in our main

manuscript that **meeting physical activity recommendations was not associated with cognitive outcomes**. Similarly, **sex differences** were minor and mostly non-significant, although boys tended to respond faster, which was consistent with the original analyses.

In summary, these continuous-variable models strengthen and extend our original findings by showing that the observed patterns are not limited to categorical group comparisons. Instead, they reflect broader linear associations particularly the negative relationship between zBMI and processing speed, and the strong positive influence of age on all aspects of performance. Taken together, these analyses support the robustness of our original conclusions and add further nuances to our understanding of how developmental and health-related factors relate to cognitive control.

Table 2. Multiple linear regression predicting accuracy for congruent stimuli

Predictors	Coefficients	CI	*p*
Age (continuous)	1.02	1.01 – 1.04	**<0.001**
MVPA (continuous)	1.00	1.00 – 1.00	0.326
zBMI (continuous)	1.01	0.99 – 1.02	0.384
Reaction time for congruent stimuli (continuous)	1.00	1.00 – 1.00	**0.011**
Female	Ref		
Male	1.03	0.99 – 1.06	0.136

In this multiple linear regression predicting accuracy for congruent stimuli (Table 2)**,** age was significantly associated with higher accuracy in the congruent condition (β = 1.02, 95% CI: 1.01–1.04, p < 0.001), confirming that older children tend to perform better. Reaction time was also significantly related to accuracy (β = 1.00, p = 0.011), suggesting a small but meaningful association between slower response times and greater accuracy perhaps reflecting more deliberate responding. Neither zBMI, MVPA, nor sex significantly predicted accuracy. These results support our previous categorical findings in the manuscript showing no significant association between nutritional status and accuracy, and that age was positively associated with accuracy for congruent stimuli. The new model confirms that nutritional status (zBMI) is not linearly related to accuracy, and age remains a robust predictor.

Table 3. Multiple linear regression predicting accuracy for incongruent stimuli

Predictors	Coefficients	CI	*p*
Age (continuous)	1.04	1.02 – 1.05	**<0.001**
MVPA (continuous)	1.00	1.00 – 1.00	0.367
Zbmi (continuous)	1.00	0.98 – 1.02	0.842
Reaction time for incongruent stimuli (continuous)	1.00	1.00 – 1.00	0.219
Female	Ref		
Male	1.03	0.99 – 1.08	0.159

In this multiple linear regression predicting accuracy for incongruent stimuli (Table 3), age again turned out to be a significant predictor (β = 1.04, 95% CI: 1.02–1.05, $p < 0.001$), indicating that older children exhibited greater cognitive control under more demanding conditions. No significant associations were found for zBMI, MVPA, reaction time, or sex. These results reinforce our original findings presented in the main manuscript that age is consistently associated with higher accuracy under both conditions, whereas nutritional status and physical activity are not significantly related with accuracy. The consistency across models strengthens confidence in age as a key developmental factor.

Table 4. Multiple linear regression predicting reaction time for congruent stimuli

Predictors	Coefficients	CI	*p*
Age (continuous)	-46.44	-59.43 – -33.44	**<0.001**
MVPA (continuous)	0.59	-0.09 – 1.27	0.090
Zbmi (continuous)	21.81	5.12 – 38.50	**0.010**
Accuracy for congruent stimuli (continuous)	133.19	19.81 – 246.57	**0.021**
Female	Ref		
Male	-57.73	-101.19–14.27	**0.009**

In this multiple linear regression predicting reaction time for congruent stimuli (Table 4), age was a strong and significant predictor of faster reaction times (β = -46.44, p < 0.001), consistent with developmental improvements. Boys responded significantly faster than girls (β = -57.73, p = 0.009), whereas higher zBMI was significantly associated with slower reaction times (β = 21.81, p = 0.010). MVPA showed a marginal (statistically non-significant) trend (p = 0.090), while greater accuracy was associated with slightly slower responses (β = 133.19, p = 0.021). This model aligns closely with our original findings presented in the main manuscript showing that underweight children responded faster than normal-weight peers, and that age and sex were significantly associated with reaction time. The continuous zBMI result adds precision to the prior categorical comparison, showing that increasing body mass is linked to slower processing speeds.

Table 5. Multiple linear regression predicting reaction time for incongruent stimuli

Predictors	Coefficients	CI	*p*
Age (continuous)	-44.70	-58.99 – -30.41	**<0.001**
MVPA (continuous)	0.46	-0.28 – 1.20	0.224
zBMI (continuous)	23.59	5.44 – 41.74	**0.011**
Accuracy for incongruent stimuli (continuous)	-58.15	-155.12 – 38.81	0.239
Female	Ref		
Male	-33.24	-80.58 –14.10	0.168

In this multiple linear regression predicting reaction time for incongruent stimuli (Table 5), age again significantly predicted faster responses (β = -44.70, p < 0.001), confirming its consistent role in cognitive speed. **Higher zBMI was significantly associated with slower reaction times** (β = 23.59, p = 0.011), replicating the effect seen in the congruent condition. Other predictors, including accuracy, MVPA, and sex, were not significant. This supports our earlier finding that **overweight children had slower reaction times** for incongruent stimuli and that older **age is associated with improved processing speed**. The new results extend our original findings presented in the main manuscript by showing a **linear trend** in the zBMI reaction time relationship across the entire spectrum, rather than just at category boundaries.
